# Supercomplex Restructuring in Heart Mitochondria of COX7A1-Deficient Mice

**DOI:** 10.3390/biom15091209

**Published:** 2025-08-22

**Authors:** Lauren Pavelich, Lucynda Pham, Paul Stemmer, Icksoo Lee, Lawrence I. Grossman, Maik Hüttemann, Tasnim Arroum

**Affiliations:** 1 Center for Molecular Medicine and Genetics, Wayne State University, Detroit, MI 48201, USA; laurenpavelich@wayne.edu (L.P.); lucynda.pham@med.wayne.edu (L.P.); lgrossman@wayne.edu (L.I.G.); 2 Department of Biochemistry, Microbiology, and Immunology, Wayne State University, Detroit, MI 48201, USA; 3 Department of Pharmaceutical Sciences, Wayne State University, Detroit, MI 48201, USA; pmstemmer@wayne.edu; 4 College of Medicine, Dankook University, Cheonan 31116, Republic of Korea; icksoolee@dankook.ac.kr

**Keywords:** cytochrome *c* oxidase, isoforms, COX7aH, COX7aL, heart

## Abstract

The role of electron transport chain supercomplexes and factors that regulate their composition in a tissue- and species-specific manner are not fully understood. Tissue-specific isoforms have been reported for cytochrome *c* oxidase (COX), which may contribute to such regulation. Therefore, we here investigated COX activity and structural organization in wild-type (WT) and COX7A1 knockout (KO) mice, which lack the heart/skeletal muscle isoform of COX subunit VIIa. COX7A1 KO mice showed a 30% reduction in total COX activity in the heart. Although the activity of COX in the monomers and I+III_2_+IV_n_ supercomplexes (SCs) remained unchanged, a marked reduction in COX dimers and unknown COX-containing species IV_x_ and IV_y_ contributed to the overall reduction in COX activity. Furthermore, we observed that COX7A2 substituted for COX7A1 in COX monomers, dimers, and all COX-containing SCs in the KO mice, indicating a compensatory mechanism to preserve COX functionality. Collectively, these results suggest that COX7A1 plays an important role in maintaining structural stability; however, they also suggest that loss of COX7A1 is compensated by its replacement with COX7A2.

## 1. Introduction

Cytochrome *c* oxidase (COX), the terminal oxidase of the mitochondrial electron transport chain (ETC), is a crucial enzyme involved in aerobic respiration and ATP synthesis. The enzyme’s complex assembly comprises multiple subunits that are expressed in a tissue-specific manner, each playing an integral role in the enzyme’s functional activity and regulatory mechanisms. Tissue-specific COX subunit isoforms could serve as a foundation for fine-tuning COX activity to meet the specific metabolic needs of different tissues. Among these subunits is COX subunit 7a (COX7A), which is comprised of distinct isoforms, with COX7A1 (the “heart-type” isoform, also called COX7AH) demonstrating tissue-specific expression, predominantly within cardiac and skeletal muscle tissue, as high-energy-demanding tissues [1]. Initial investigations concentrated on elucidating the characteristics of the COX7A1 gene [2], delineating its genomic architecture and promoter regulation while revealing its structural homology to the ubiquitously expressed COX7A2 (the “liver-type” isoform, also known as COX7AL) and emphasizing their distinct gene regulation [3,4,5]. Despite their structural similarity, these isoforms are encoded by separate genes and controlled by distinct transcriptional mechanisms, reflecting their specialized roles. Comparative genomic investigations further highlighted the evolutionary divergence of these isoforms, indicating functional specialization influenced by adaptive evolutionary pressure in primates, with an accelerated nonsynonymous substitution rate in the COX7A gene family—COX7A1, COX7A2, and COX7AR (also known as COX7A2L or SCAF1), aligning with the evolution of tissue-specific functions [6].

We have previously characterized the role of *Cox7a1* in a knockout mouse model, highlighting its important role in maintaining cardiac functionality. COX7A1 loss caused a decrease in total COX activity and manifestation of dilated cardiomyopathy in 6-week-old mice, which improved over time [7]. In addition, we found that COX7A1 knockout mice incorporate the liver-type isoform (COX7A2) as a compensatory mechanism. In addition, we found that COX7A1 loss leads to reduced skeletal muscle bioenergetics and causes exercise intolerance [8]. Investigations into isoform switching between COX7A1 and COX7A2 have further illuminated the role of COX7A1 in cardiac and skeletal muscle, showing a developmental transition from liver-type to heart/muscle-specific isoforms of both COX6A and COX7A during early postnatal muscle development in both human and mouse skeletal muscle, with the heart/muscle-specific isoform becoming predominant by 3 months of age in infants and 4 weeks in mice [9]. However, the authors further show that biopsy studies in patients with reversible infantile respiratory chain deficiency showed that this isoform switch does not directly contribute to the onset or recovery of the disease [9]. It should be noted that commonly used in vitro models, such as the C_2_C_12_ myoblast differentiation model, fail to fully recapitulate this switch, exhibiting only limited expression of the muscle-specific isoform, thus making them suboptimal for studying the isoform switch and adaptation of muscle cells [10,11].

In this study, we aimed to investigate how deletion of the COX7A heart-specific isoform subunit affects COX activity and mitochondrial supercomplex assembly, with a particular focus on isoform switching of COX7A1 and two subunits in cardiac mitochondria in female mice.

## 2. Materials and Methods

### 2.1. Generation of Cytochrome c Oxidase Subunit 7a Isoform 1 (Heart-Type) Knockout Mice

The generation of knockout mice by replacement of all three *Cox7a1* exons with the *neo* cassette was described in detail in [7].

### 2.2. Heart Tissue Homogenization and Mitochondria Isolation

Heart tissue was homogenized using gentleMACs Dissociator (Miltenyi Biotec, Bergisch Gladbach, Germany, Cat. No 130-093-235) with protocol m_lung_02.01 in 2 mL of mitochondrial isolation buffer (MIB: 200 mM sucrose, 10 mM Tris, 1 mM EGTA, pH 7.4 adjusted with 1 M HEPES solution, Gibco, cat. No. 15630080) in gentleMACs C Tubes (Miltenyi Biotec, Cat. No. 130-093-237). Heat-activated sodium vanadate was added to the homogenization buffer at a final conentration of 5mM. The entire process was performed on ice to help preserve protein integrity and prevent dephosphorylation. To further break down the tissue, 0.2 mg of Subtilisin A (Protease) (Sigma, Burlington, MA, USA, Cat. No. P5380) was added to the tissue homogenate and incubated at 4 °C for 10 min with gentle shaking. To enrich the mitochondrial fraction, we used a series of filtration steps. The filters were pre-wet with MIB. First, a 40 μm filter (pluriSelect, Cat. No. 43-50040-50) was used to remove larger cellular debris and nuclei. The flowthrough containing mitochondria and other organelles was then collected and subjected to two centrifugations at medium speed (9000× *g*, 15 min, 4 °C) to precipitate the crude mitochondrial fraction and further remove contaminations. Mitochondrial fractions were washed with MIB supplemented with 2 mM phenylmethylsulphonylfluoride, 5 mM heat-activated sodium vanadate, and Protease Inhibitor Cocktail (Sigma, Cat. No. P8849). Protein concentration was determined using the DC protein assay kit (Bio-Rad, Hercules, CA, USA, cat. no. 5000111), according to the manufacturer’s protocol.

### 2.3. Outer Mitochondrial Protein Digestion with Proteinase K

Isolated mitochondria were digested with Proteinase K solution (QIAGEN, Hilden, Germany, cat. No. 1018332) to remove the cytosolic and outer mitochondrial membrane proteins. Eighty µg of mitochondria were resuspended in 1 mL of MIB plus Proteinase K solution at a final concentration of 1mM and incubated at room temperature for 45 min, with gentle shaking. To remove the Proteinase K, Protease Inhibitor Cocktail (1:100) was added, and the solution was centrifuged at 16,900× *g* for 15 min at 4 °C to collect the mitochondrial pellet. The pellet was then washed 3 times with fresh MIB. Western blot analysis of the proteinase K treatment is presented in Supplementary Figure S3.

### 2.4. Mitochondrial Membrane Solubilization for Blue Native-PAGE

The Blue Native-PAGE (BN-PAGE) protocol was adapted from [12,13]. All components were used from the NativePAGE™ Sample Prep Kit (ThermoFisher Scientific, Waltham, MA, USA, cat. no. BN2008) including NativePAGE™ 4× sample buffer, NativePAGE™ 5% G-250 sample additive, except the digitonin (ThermoFisher Scientific, cat. no. 407565000), which was diluted to 5%. Briefly, for solubilization of crude mitochondria, we used NativePAGE™ 4× sample buffer supplemented with digitonin (5%) to reach a final concentration of 1.5% (digitonin/protein ratio of 6 *g*/*g*). This mixture was incubated on ice for 20 min, mixing every 5 min to ensure complete solubilization of the mitochondria. The mixture was then centrifuged at 4 °C at 17,000× *g* for 20 min. Following centrifugation, 0.5% G-250 sample additive was added to the supernatant. Subsequently, 75 μg of these samples were loaded onto 3–12% native gradient gels (Invitrogen, Carlsbad, CA, USA cat. no. BN1001BOX). Electrophoresis was conducted at 4 °C, applying 150 V until the proteins entered 1/3 into the gel (~30 min); at this point, the light-blue cathode buffer was changed to clear cathode buffer (50 mM Tricine, 15 mM Bis-Tris, pH 7.0). The voltage was then increased to 200 V until the desired separation was achieved (~1 h). The gels to be used for Western blot analyses were stained with GEL Blue (G-Biosciences, St. Louis, MO, USA, cat. no. 786-35G) for 30 min. The gel was washed 2 × 5 min with water before being imaged. The mitochondrial proteins on the gel were then transferred from the native gel to a PVDF membrane (BioRad, cat. no. 1620177) via a wet transfer at 10 V overnight. To help transfer large proteins, 0.05% SDS was added to the transfer buffer (25 mM Tris base 192 mM glycine, 20% methanol, pH adjusted to 8.3). Thirty µg of the mitochondrial sample from BN-PAGE were run on an SDS PAGE, followed by Western blot, to determine the purity of the inner mitochondrial membrane proteins (Supplementary Figure S3).

### 2.5. Mitochondrial Native Protein In-Gel Activity Assay (IGA)

After the BN-PAGE, we conducted an in-gel activity assay (IGA), as described [13]. Briefly, gels were incubated in 10 mL of the respective complex assay buffer at room temperature. COX IGAs were completed first, followed by complex I (CI) activity assays performed on the same gels. Each reaction was carried out at room temperature for 1 h with gentle shaking. Gels were washed 2 × 10 min in water and then imaged. The gels were washed in water overnight at 4 °C and then imaged again. To avoid saturated signals, pictures obtained after one hour of incubation were used for quantification.

### 2.6. Western Blot Analysis

To remove the Coomassie from the membrane, a 15 min methanol wash step was performed. Membranes were washed 2 × 5 min with water and 2 × 5 min with TBST to remove methanol. Membranes were blocked with a blocking reagent (5% bovine serum albumin (BSA), 1% Tris-buffered saline with Tween-20: TBST, 0.1% Tween- 20) for 45 min at room temperature. Next, we added the primary antibody (Table 1) with gentle shaking for 90 min at room temperature or overnight at 4 °C. The membranes were then washed two times for 5 min with TBST. Finally, membranes were incubated with the secondary antibody for 1 h at room temperature (Table 1), overlaid with Pierce™ ECL Western Blotting Substrate (ThermoFisher, cat. no. 32106), and signals were recorded on a ChemiDoc MP imaging system.

### 2.7. Mass Spectrometry and Proteomic Analysis

Following BN-PAGE, gel pieces were excised and peptides were prepared using the in-tryptic digestion method, following the manufacturer’s protocol (Thermo Scientific™ Waltham, MA USA, cat. no. 89871). Peptide samples were analyzed on a Thermo Scientific Orbitrap Eclipse Tribrid mass spectrometer, operated in data-dependent acquisition (DDA) mode. Raw data files were processed using the MaxQuant platform (v2.6.8.0) and searched against the UniProt database for Mus musculus (https://www.uniprot.org/proteomes/UP000000589, accessed on 25 February 2025). For stoichiometric analysis, the peptide intensity signals for each band were used, normalized to a core subunit in the studied complex.

### 2.8. Statistical Analyses

All data are presented as means ± SEM (standard error of the mean) values. We conducted an unpaired *t*-test. Statistical analyses were conducted with GraphPad Prism version 10.0.0, GraphPad Software, Boston, MA, USA, with significance set at *p* ≤ 0.05.

## 3. Results

### 3.1. COX7A1 KO Decreases COX Activity and Amount in Select Supercomplexes

**Figure 1:**
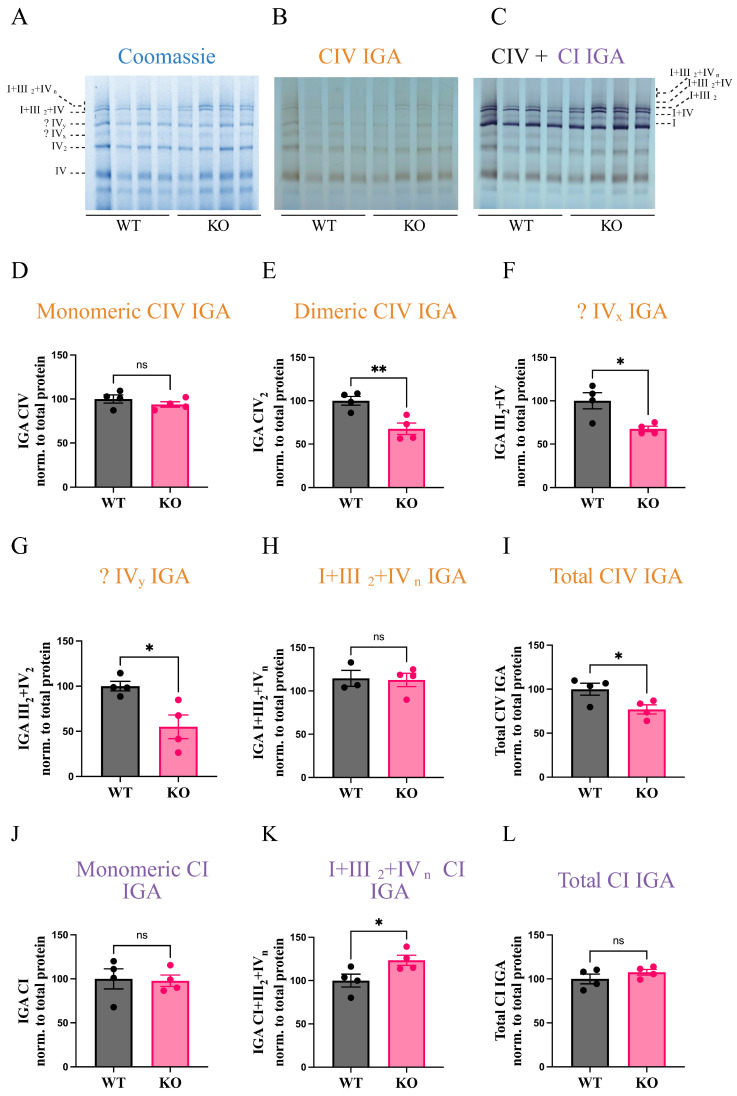
COX7A1 knockout decreases COX activity. BN-Page was performed and was followed by in-gel activity reactions for CIV and CI. Brown and violet bands indicate CIV and CI activity, respectively. (**A**) Coomassie gel before IGA was performed. (**B**) CIV IGA. (**C**) CIV+CI IGA. Uncropped images for (**A**–**C**) are found in Supplementary Figures S4 and S5. (**D**) Quantification of monomeric CIV IGA. (**E**) Quantification of CIV_2_ IGA. (**F**) Quantification of IV_x_ CIV-IGA. (**G**) Quantification of IV_y_ CIV-IGA. (**H**) Quantification of I+III_2_+IV_n_ CIV-IGA. (**I**) Quantification of total CIV IGA. (**J**) Quantification of monomeric CI IGA. (**K**) Quantification of I+III_2_+IV_n_ CI-IGA. (**L**) Quantification of total CI IGA normalized to total protein. Data was from four female animals per group. Data are represented as means ± standard error. **, *p* < 0.01; *, *p* < 0.05, based on unpaired *t*-test. The orange color is for complex IV in-gel activity and violet color for complex I in-gel activity.

To study the effect of knockout of the heart/skeletal muscle-specific COX subunit 7A1 on supercomplex composition, we investigated female WT and *Cox7a1* KO mice to analyze COX activity and supercomplex assembly changes in the heart. To explore potential alterations in the activity and composition of COX monomers, dimers, and supercomplexes (SCs), we employed Blue-Native PAGE (BN-PAGE) followed by in-gel activity (IGA) assays for COX and complex I (CI), along with immunoblotting of various ETC complexes. BN-PAGE, in combination with mild solubilization of mitochondrial membrane proteins using digitonin, preserves protein–protein interactions and maintains native complex structure and activity. This enabled us to assess structural and functional rearrangements of COX-containing SCs. Interestingly, IGA analysis of COX revealed that COX monomer activity remained unchanged (Figure 1B,D). However, activity within COX dimers (IV_2_) and unknown CIV species that run at similar electrophoretic mobility as the III_2_+IV and III_2_+IV_2_ SCs (annotated as IV_x_ and IV_y_) exhibited significant reductions (Figure 1E–G). When considering overall COX activity across all complexes, we observed a total decrease of 30% (Figure 1I). Interestingly, this reduction was not evenly distributed across COX-containing complexes; rather, select subpopulations, such as COX dimers and IV_x_ and IV_y_ species, contributed disproportionately to the observed decline in COX activity. We also investigated CI in-gel activity (Figure 1C). Our results showed that CI monomer activity and CI-associated SC activity remained unchanged (Figure 1J and Figure S1B,C). The only exception was SC I+III_2_+IV_n_, which exhibited a ~30% increase in activity (Figure 1K). Notably, this was the only SC where COX activity was preserved and did not show a reduction (Figure 1H). This suggests that in the absence of COX7A1, respiratory chain complexes undergo selective rearrangements, with certain SCs compensating to maintain respiratory chain integrity.

### 3.2. Proteomic Profiling Shows Distinct Distribution of the COX7A1 and COX7A2 in WT and KO Mice

**Figure 2:**
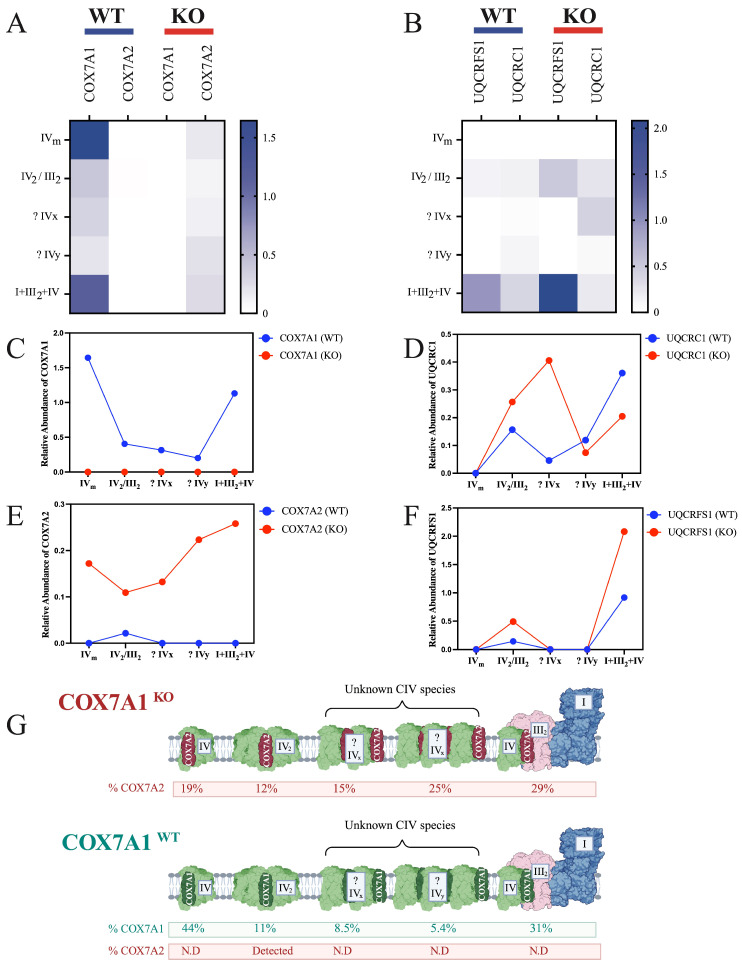
Structural compensation and redistribution of COX7A2 in COX7A1 knockout mice. (**A**) BN-PAGE followed by proteomic analysis, illustrating the relative distribution of COX7A1 and COX7A2. The total intensity was normalized to the COXIV subunit within each gel band. (**B**) BN-PAGE followed by proteomic analysis, illustrating the relative distribution of UQCRFS1 and UQCRC1. The total intensity was normalized to the UQCRC2 subunit within each gel band. (**C**,**E**) Relative abundance comparison of COX7A1 and COX7A2, based on data shown in (**A**). (**D**,**F**) Relative abundance comparison of UQCRC1 and UQCRFS1, based on data shown in (**B**). (**G**) Summary schematic of data derived from (**A**–**F**). Not detected annotated as N.D.

Mass spectrometry, following BN-PAGE and band excision, was used to investigate the distribution profiles of COX7A1 and COX7A2 across different mitochondrial complexes in COX7A1 WT and KO mice. In WT mice, COX7A1 is primarily found in monomers (44%) and dimers (11%), in addition to its presence in IV_x_, IV_y_, and I+III_2_+IV SCs. In contrast, COX7A2 was notably reduced in WT mice, accounting for only 5% of the total COX7A levels found solely in COX-dimeric assemblies (Figure 2A–G). Conversely, in COX7A1 KO mice, COX7A1 was absent as anticipated, while COX7A2 appeared in monomers (19%), dimers (12%), IV_x_ and IV_y_ species (15% and 25%), and 29% in the I+III_2_+IV SC (Figure 2A–G). This distribution suggests that COX7A2 compensates for the lack of COX7A1 in the KO animals, leading to a reorganization of COX. Although the redistribution of COX7A2 highlights its structural importance in stabilizing COX, these structural adaptations were insufficient to fully restore COX activity (Figure 1I).

**Figure 3:**
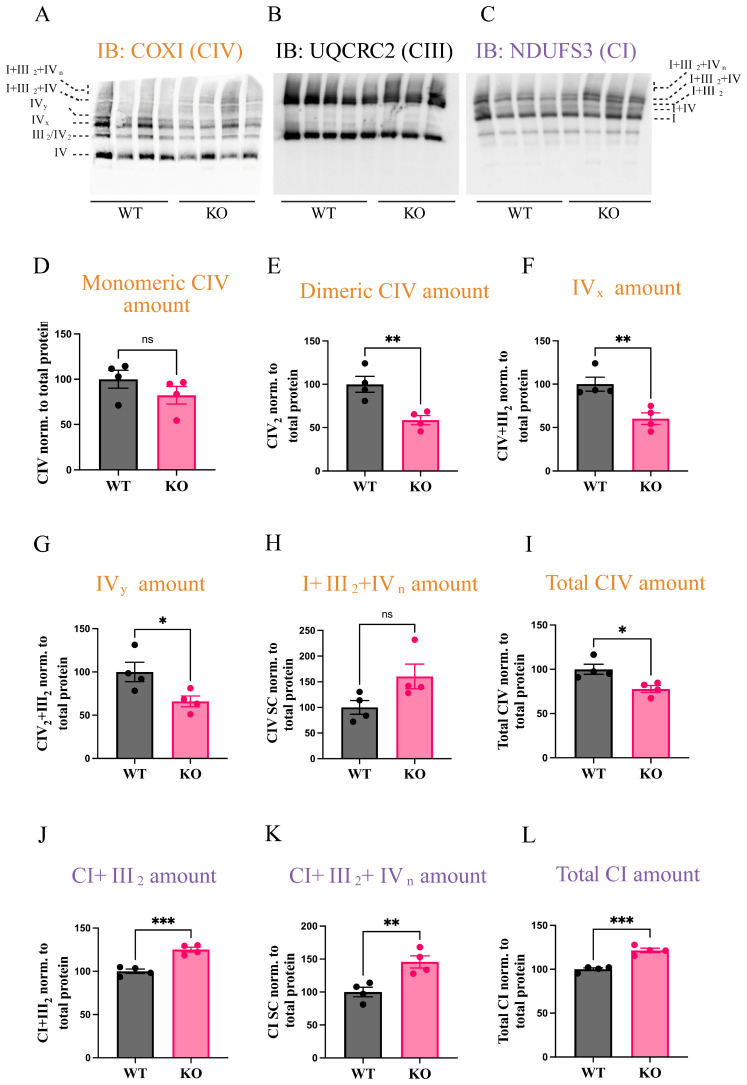
COX7A1 KO decreases COX amount. BN-Page was performed and was followed by immunoblotting. (**A**) Western blot following anti-COXI (CIV). (**B**) Western blot following anti-UQCRC2 (CIII). (**C**) Western blot following anti-NDUFS3 (CI). Uncropped western blots for (**A**–**C**) are found in Supplementary Figures S6 and S7. (**D**) Quantification of monomeric CIV amount. (**E**) Quantification of CIV_2_ amount. (**F**) Quantification of CIV amount in IV_x_. (**G**) Quantification of CIV amount in IV_y_. (**H**) Quantification of CIV amount in I+III_2_+IV_n_. (**I**) Quantification of total CIV amount. (**J**) Quantification of CI amount in I+III_2_. (**K**) Quantification of CI amount in I+III_2_+IV_n_. (**L**) Quantification of total CI. Data was from four female animals per group. Data are represented as means ± standard error. ***, *p* < 0.001; **, *p* < 0.01; *, *p* < 0.05, based on unpaired *t*-test. Orange is for complex IV immunoblot quantification and violet is for complex I immunoblot quantification.

We did not detect SCAF1 in our COX complexome analysis, despite observing a signal at the same level as III_2_+IV_1-2_ (Figure 1A,B, Figure 3A,B and Figure S2). This signal was notably more evident when using COX antibodies compared to CIII antibodies, and when the image was overexposed. The mice used have a C57BL/6 background that is known to lack SCAF1. Immunoblot with SCAF1 and UQCRC1 is presented in Supplementary Figure S2.

We next assessed the stoichiometric distribution of complex III (*bc_1_*-complex; Figure 2B) by examining representative subunits indicative of specific assembly stages. UQCRC1, a core subunit involved in all stages of complex III biogenesis, served as an indicator for the presence of complex III in the selected bands, while UQCRFS1 was used as a marker of mature dimeric CIII_2_. We differentiated between pre-III_2_ (immature) and mature III_2_ complexes in both WT and KO animals. Specifically, the absence of UQCRFS1 signified pre-III_2_ structures, whereas its presence confirmed complete dimerization. In Figure 2B, we can see dimerization of CIII in III_2_ and supercomplex bands, while immature pre-III_2_ complexes were identified running at the same level as the IV_x_ and IV_y_ bands, where no UQCRFS1 signal was detected. However, due to the lack of SCAF1 in these animals, these signals are not indicative of an assembled III_2_+IV complex, and may be due to disassembly of higher I+III_2_+IV SC due to detergent treatment.

We next examined potential changes in ETC content using immunoblotting. Following BN-PAGE separation, we performed immunoblotting for COX (COXI), CIII (UQCRC2), and CI (NDUFS3) (Figure 3A–C). Consistent with COX IGA findings, there was no significant change in monomeric COX levels or I+III_2_+IV_n_ SCs (Figure 3D,H). However, decreases were observed in dimeric COX, IV_x_, and IV_y_ (Figure 3E–G). Additionally, we investigated complex I assembly, noting an increase in CI within I+III_2_ and I+III_2_+IV_n_ supercomplexes (Figure 3J–L). Quantitative analysis revealed a 20% increase in total CI levels (Figure 3L) and a 30% decrease in total COX levels (Figure 3I). Further examination of complex III showed no alteration in III_2_, I+III_2_, I+III_2_+IV_n_, or total CIII content (Supplementary Figure S1G,H,K).

## 4. Discussion

In this study, we investigated the assembly and redistribution of mitochondrial complexes and SCs in the hearts of COX7A1 knockout mice. Using in-gel activity analysis of COX, we gained new insights into how a selective reduction of activity in COX-containing complexes took place in COX7A1-deficient mouse hearts. Recently, García-Poyatos et al. [14] showed that loss of *Cox7a1* in zebrafish leads to a severe reduction in COX homodimerization and the assembly of higher-order supercomplexes, without affecting the levels of monomeric COX. This disruption was accompanied by a marked decrease in total COX activity in *Cox7a1*-deficient zebrafish skeletal muscle. In our study, the enzymatic activity of monomeric COX remained unchanged; however, the activity associated with COX dimers and COX containing IV_x_ and IV_y_ species was significantly reduced. This suggests that the loss of COX7A1 may not impact the catalytic function of monomeric COX; rather, it could play a role in stabilizing COX homodimerization and higher-order structures involving COX. However, the decrease observed in the mouse model was not as severe as reported in the zebrafish knockout model [14]. Moreover, homodimeric COX exhibits higher enzymatic activity in the zebrafish model than its monomeric counterpart. The latter contradicts previous biochemical and structural studies conducted in mammalian tissue and in vitro enzymatic studies; we and others have shown that COX inhibition through allosteric regulation and post-translational modifications is disrupted under stress conditions, likely by Ca^2+^-activated dephosphorylation of COX, which causes COX to transition into its monomeric form—proposed as the more active form of COX [13,15,16,17].

Our results show that the activity of monomeric CI and CI-containing supercomplexes remained largely unchanged between WT and *Cox7a1* knockouts. Interestingly, the only notable exception was SCI+III_2_+IV_n_, which exhibited a ~30% increase in CI activity. I+III_2_+IV_n_ is the COX-containing SC in which COX activity remained preserved and did not decline. These findings suggest that, in the absence of COX7A1, a compensatory rearrangement within the ETC takes place, favoring the assembly of CI-containing SCs, potentially to rescue some of the respiratory dysfunction. This stabilization of higher-order CI-containing SCs was observed in other reports as a response to ETC defects [18,19]. When COX7A1 is absent, selective rearrangements happen within respiratory chain complexes, allowing specific supercomplexes to compensate and maintain respiratory chain integrity. Previously, we demonstrated that *Cox7a1* knockout mice showed increased incorporation of the “liver-type” isoform COX7A2 into the cardiac COX holoenzyme [8]. Rodents, like humans, undergo a developmental switch from “liver-type” COX7A2 to the “heart-type” COX7A1 during postnatal maturation [9]. In the adult stage, COX7A1 is the predominant form. However, in *Cox7a1* knockout mice, we see that COX7A2 is upregulated and is compensating for COX7A1 loss. In the present study, we expanded upon these findings using mass spectrometry following BN-PAGE analysis, revealing that COX7A2 is incorporated into all COX populations, including monomers, dimers, and higher-order supercomplexes in the mitochondria lacking COX7A1. The redistribution of COX in the KO model may help increase the enzymatic turnover rate through monomerization, leading to increased respiration that compensates for COX7A1 loss, resulting in the preference of the more active monomeric COX after the loss of COX7A1.

Previously, we found that COX7A1 KO was accompanied by an unexpected elevation in tissue ATP levels [7]. Notably, the overall reduction in COX dimerization observed in the knockout mice may help explain the elevated ATP levels, as dimeric COX has been associated with increased inhibitory phosphorylation and lower activity in vitro [20] and in pathological conditions in liver tissue [13]. COX phosphorylation was linked to increased dimer formation and decreased enzymatic activity, and potentially caused allosteric constraints that limit the turnover rate of the enzyme. Moreover, elevated cAMP levels, which promote COX phosphorylation [17], are known to favor dimer formation and suppress respiratory activity [17,21], thus limiting mitochondrial output and reactive oxygen species (ROS) generation [20]. In this context, dynamic regulation of COX dimerization may serve as a protective mechanism in high-energy-demanding tissues like the heart and skeletal muscle, helping to fine-tune respiration and minimize oxidative stress and cellular damage in tissues lacking COX7A1.

The interplay between the different isoforms needs to be further investigated and validated through additional techniques not reliant on detergent enrichment, as previous studies have explored how different detergents influence the dimerization of COX. Biochemical and structural studies have shown, for instance, that sodium cholate promotes the formation of COX dimers by binding to its dimer interface and thereby stabilizing its dimeric form [20,22]. In addition, cardiolipin was found to promote COX dimer formation [23]. This suggests that removing the cardiolipin-COX interface through detergents could destabilize dimers, increase the monomeric form of COX, and affect the dimeric turnover rate. Therefore, changes in the lipid composition and their interaction with mitochondrial ETC subunits could influence the complex and SC populations and their activity. In this study, we used digitonin, the mildest non-ionic detergent that produces a heterogeneous mix of monomeric, dimeric, and higher-order structures of COX. It frees COX from the lipid bilayer, which by itself may decrease dimerization of COX observed in the native gel analysis. However, at the moment, it is the best detergent used for mitochondrial SC enrichment.

Another subunit of the COX7A family is SCAF1 (also known as COX7AR or COX7A2L). Due to the mouse C57BL/6 background, we did not detect SCAF1 in our mass spectrometry data, confirming previous reports [24,25]. Interestingly, we observed the presence of bands with a similar electrophoretic mobility of III_2_+IV_1-2._ This signal corresponds to unknown higher-order COX species annotated as IV_x_ and IV_y_ in this report. The lack of SCAF1 leads to loss of III_2_+IV SC formation. Also, the III_2_+IV_2_ SC was not reported in mammals, and it has been shown that a single copy of subunit 9 spans both CIII monomers, blocking the binding of a second SCAF1 molecule and thereby preventing the assembly of the III_2_+IV_2_ supercomplex [25]. Therefore, the observed COX species above the COX dimers are not characterized yet. These COX-containing species were also observed previously, in [24].

We also detected CIII at the same position as the IV_x_ and IV_y_ bands, but CIII was not fully mature and, notably lacked UQCRFS1, which is found in the mature III_2_ complex [26]. Full-length SCAF1 is essential for promoting the formation of the III_2_+IV SC by effectively tethering complexes III and IV [27], which is important for I+III_2_+IV_n_ diversity [28], and it has been shown to regulate CIII biogenesis. However, SCAF1 did not affect mitochondrial respiration in the human cell culture system [29]. Additionally, SCAF1 and COX7A1/2 have been shown to form distinct types of respirasomes, with SCAF1-respirasome being preferred under glycolytic conditions and COX7A1/2 being more favorable for promoting OXPHOS when pyruvate dehydrogenase is activated [30].

Finally, previous studies using *Cox7a1* knockout mice have only examined male animals, reporting a reduction of approximately 50% in COX activity [7]. Although we observe a similar reduction in COX activity in female animals (this study), it is not as pronounced, with an overall reduction of 30% in *Cox7a1* knockout, suggesting that there are sex-specific modulators of ETC function that merit further investigation. Previous research investigating mitochondrial biogenesis and function in cardiac tissue has yielded varied findings regarding sex-related differences in mitochondrial function. Some studies have reported reduced mitochondrial DNA content and decreased mitochondrial function in female hearts compared to males [31]. In contrast, others have found that female hearts typically exhibit higher mitochondrial state 3 respiration, compared to male hearts [32]. Further investigations into hormonal regulation and other sex-specific mechanisms that influence cardiac mitochondrial function are warranted.

## 5. Conclusions

In conclusion, our study demonstrates that loss of COX7A1 in female mouse hearts specifically impairs COX activity within dimeric and higher-order supercomplexes, while monomeric COX remains functionally intact. The observed compensatory increase in COX7A2 incorporation in *Cox7a1* knockout, along with selective rearrangements favoring CI-containing supercomplexes, appears to help mitigate respiratory malfunction. Moreover, sex-specific differences in COX activity reduction underscore the need for further investigation into hormonal and genetic modulators of mitochondrial function.

## Figures and Tables

**Table 1:** List of antibodies used in this study.

Antibody	Dilution	µg/mL Amount	Company	Product ID
MT-CO1 (COX1)	1:1000	1	Thermofisher	PA5-68016
UQCRC1	1:2000	0.8	Proteintech	14742-1-AP
NDUFS3	1:1000	1	Abcam	17D95
COX7A2L (SCAF1)	1:1000	0.350	Proteintech	11416-1-AP
Anti-Rabbit IgG HRP-linked	1:5000	0.077	Cell Signaling Technology	7074S
Anti-Mouse IgG HRP-linked	1:8000	0.184	Cell Signaling Technology	7076S
Anti-Rabbit IgG StarBright Blue 700	1:2500	-	BioRad	12004162
Anti-Mouse IgG StarBright Blue 520	1:5000	-	BioRad	12005867

## Data Availability

The original contributions presented in this study are included in the article and Supplementary Materials. Further inquiries can be directed to the corresponding authors.

## Supplementary Materials

The following supporting information can be downloaded at: https://www.mdpi.com/article/10.3390/biom15091209/s1, Figure S1: Quantification of IGA and BN-Page; Figure S2: BN-Page with SCAF1; Figure S3: Proteinase K treatment quality control; Figure S4. Uncropped images for Figure 1B,C. CIV IGA, and CIV+CI IGA. Images were taken after overnight incubation and on a phone. Total of 4 biological replicates per group. Sample group and lane assignments: WT 1-4, COX7aH KO 5-8; Figure S5. Uncropped images for Figure 1A–C. Uncropped Coomassie and CIV IGA, and CIV+CI IGA. Images were taken after 1 h of incubation and on a Chemidoc imager and used for IGA quantification and normalization. Total of 4 biological replicates per group. Sample group and lane assignments: WT 1-4, COX7aH KO 5-8; Figure S6. Uncropped images for Figure 3A–C. Uncropped immunoblots of COXI, UQCRC2, and NDUFS3. Total of 4 biological replicates per group. Sample group and lane assignments: WT 1-4, COX7aH KO 5-8. Lane 9 was a rat heart used as a positive control for the Q-respirasome; Figure S7. Uncropped images for Figure 3. Uncropped Coomassie blot used for total protein normalization of UQCRC2 (left), COXI (right), and NDUFS3 (right). WT 1-4, COX7aH KO 5-8. Lane 9 was a rat heart used as a positive control for the Q-respirasome; Figure S8. Uncropped images for Supplementary Figure S2A,B uncropped UQCRC1 and SCAF1. Sample group and lane assignments: WT 1-4, COX7aH KO 5-8. Lane 9 was a rat heart used as a positive control for III2+IV SC; Figure S9. Uncropped images for Supplementary Figure S3.

## Abbreviations

COX	cytochrome *c* oxidase
COX7A1/COX7AH	cytochrome *c* oxidase subunit 7a, heart/skeletal muscle-type
COX7A2/COX7AL	cytochrome *c* oxidase subunit 7a, liver-type (ubiquitous)
SCAF1/COX7AR/COX7A2L	supercomplex associated factor 1
ETC	electron transport chain
SCs	supercomplexes
BN-PAGE	blue native polyacrylamide gel electrophoresis
IGA	in-gel activity assay
CI/CIII/CIV	complexes I, III, and IV of the ETC
KO	knockout
OCR	oxygen consumption rate
ROS	reactive oxygen species
SDS-PAGE	sodium dodecyl sulfate–polyacrylamide gel electrophoresis
TCA	tricarboxylic acid cycle
WT	wild-type
