# Correction: Pavelich et al. Supercomplex Restructuring in Heart Mitochondria of COX7A1-Deficient Mice. *Biomolecules* 2025, *15*, 1209

**DOI:** 10.3390/biom16020210

**Published:** 2026-01-29

**Authors:** Lauren Pavelich, Lucynda Pham, Paul Stemmer, Icksoo Lee, Lawrence I. Grossman, Maik Hüttemann, Tasnim Arroum

**Affiliations:** 1 Center for Molecular Medicine and Genetics, Wayne State University, Detroit, MI 48201, USA; laurenpavelich@wayne.edu (L.P.); lucynda.pham@med.wayne.edu (L.P.); lgrossman@wayne.edu (L.I.G.); 2 Department of Biochemistry, Microbiology, and Immunology, Wayne State University, Detroit, MI 48201, USA; 3 Department of Pharmaceutical Sciences, Wayne State University, Detroit, MI 48201, USA; pmstemmer@wayne.edu; 4 College of Medicine, Dankook University, Cheonan 31116, Republic of Korea; icksoolee@dankook.ac.kr

In the original publication [1], there was a mistake in Figure 2 as published. Figure 2D,F were duplicated in the final published version. The figure was correct in the initial manuscript, but was accidentally misplaced during the revision process and this error went undetected before publication. The corrected Figure 2 appears below. 

The authors state that the scientific conclusions are unaffected. This correction was approved by the Academic Editor. The original publication has also been updated.

## Figures and Tables

**Figure 2:**
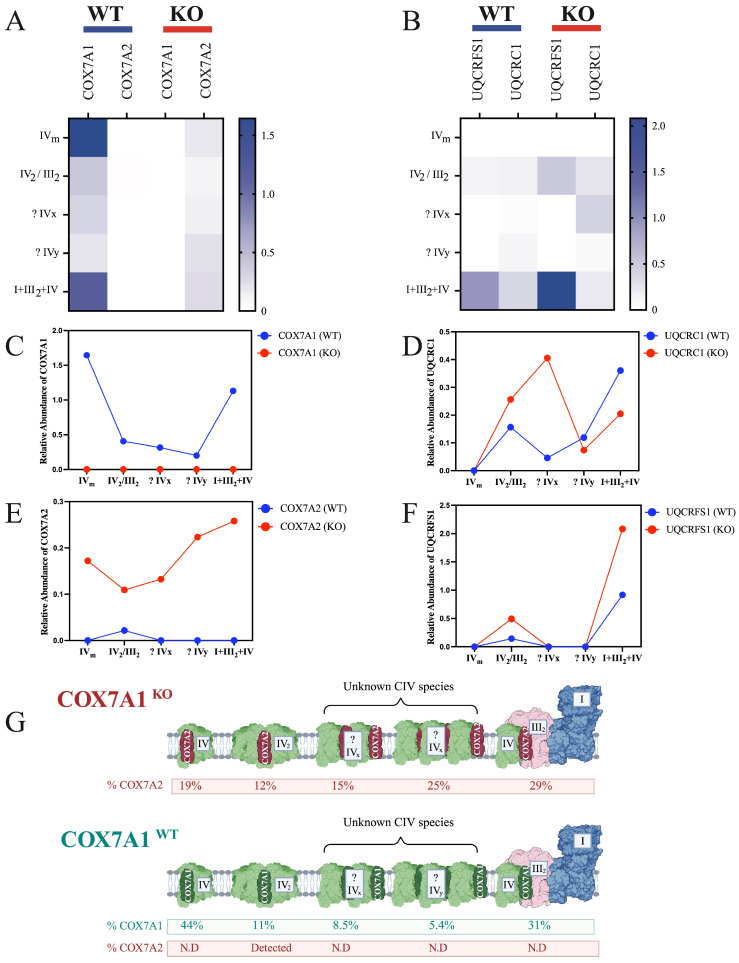
Structural compensation and redistribution of COX7A2 in COX7A1 knockout mice. (**A**) BN-PAGE followed by proteomic analysis, illustrating the relative distribution of COX7A1 and COX7A2. The total intensity was normalized to the COXIV subunit within each gel band. (**B**) BN-PAGE followed by proteomic analysis, illustrating the relative distribution of UQCRFS1 and UQCRC1. The total intensity was normalized to the UQCRC2 subunit within each gel band. (**C**,**E**) Relative abundance comparison of COX7A1 and COX7A2, based on data shown in (**A**). (**D**,**F**) Relative abundance comparison of UQCRC1 and UQCRFS1, based on data shown in (**B**). (**G**) Summary schematic of data derived from (**A**–**F**). Not detected annotated as N.D.
