# Supplementary material for: Supercomplex Restructuring in Heart Mitochondria of COX7A1-Deficient Mice
Source: Biomolecules. 2025 Aug 22;15(9):1209. doi: 10.3390/biom15091209 (PMC12467167; doi:10.3390/biom15091209)
Supplement: Supplementary file 1 [file biomolecules-15-01209-s001.zip › biomolecules-3763549-supplementary.pdf]

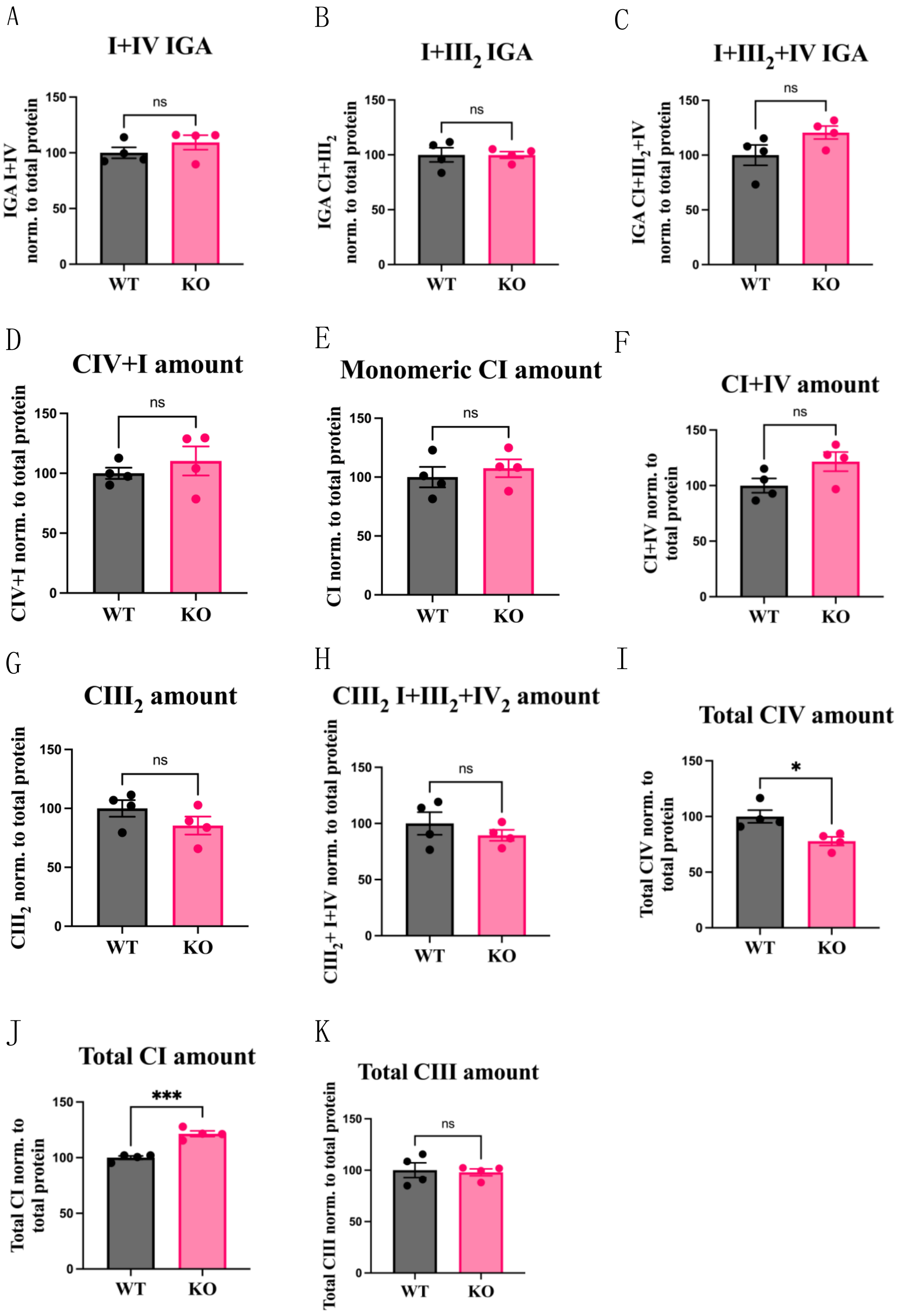
A
I+IV IGA
IGA I+IV norm. to total protein
ns
WT
KO
B
I+III2 IGA
IGA CI+III2 norm. to total protein
ns
WT
KO
C
I+III2+IV IGA
IGA CI+III2+IV norm. to total protein
ns
WT
KO
D
CIV+I amount
CIV+I norm. to total protein
ns
WT
KO
E
Monomeric CI amount
CI norm. to total protein
ns
WT
KO
F
CI+IV amount
CI+IV norm. to total protein
ns
WT
KO
G
CIII2 amount
CIII2 norm. to total protein
ns
WT
KO
H
CIII2 I+III2+IV2 amount
CIII2+ I+IV norm. to total protein
ns
WT
KO
I
Total CIV amount
Total CIV norm. to total protein
*
WT
KO
J
Total CI amount
Total CI norm. to total protein
***
WT
KO
K
Total CIII amount
Total CIII norm. to total protein
ns
WT
KO

Figure S1. Quantification of IGA and BN-Page. (**A**) quantification of I+IV CIV, IGA, (**B**) quantification of I+III$_2$ CI IGA, (**C**) quantification of I+IV CI IGA, (**D**) quantification of I+IV CIV amount, (**E**) quantification of monomeric CI amount, (**F**) quantification of I+IV CI amount, (**G**) quantification of III$_2$ amount, (**H**) quantification of I+III$_2$+IV$_n$ CIII amount, (**I**) total CIV amount, (**J**) total CI amount, (**K**) total CIII amount normalized to total protein. Data was from 4 animals per group. Data are represented as means ± standard error. ****, $p < 0.0001$; ***, $p < 0.001$; **, $p < 0.01$; *, $p < 0.05$ based on unpaired t-test.

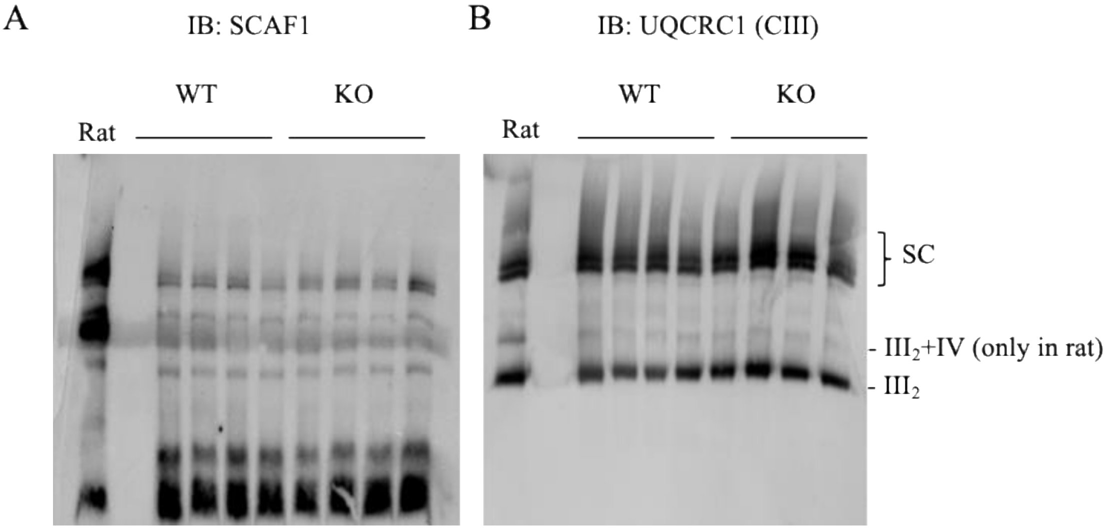

Figure S2. BN-Page with SCAF1 and UQCRC1. BN-Page was performed and followed by immunoblotting. (**A**) Immunoblot of SCAFI (COX7A2L). (**B**) Immunoblot of UQCRC1. Rattus norvegicus was used as positive control for SCAFI and III$_2$+IV signal.

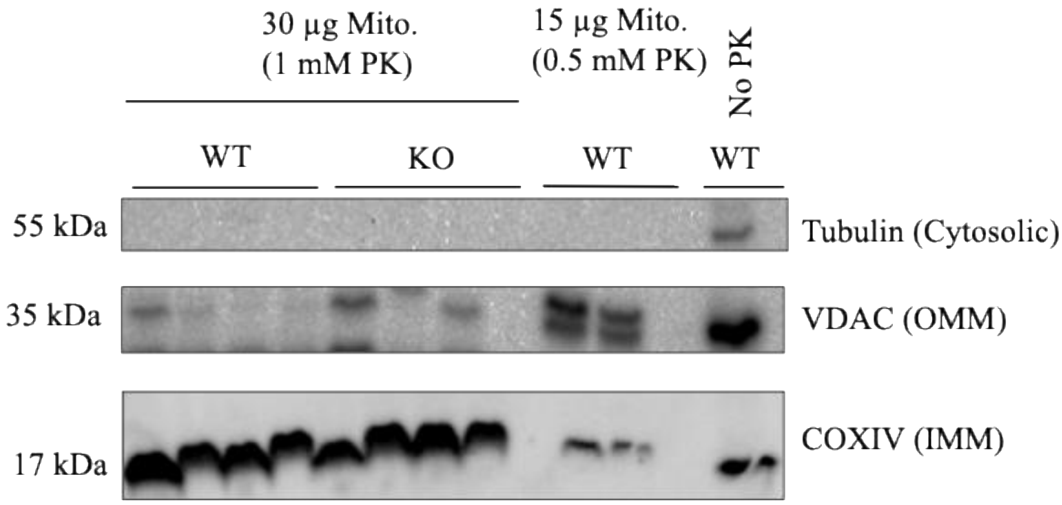

Figure S3. Proteinase K treatment quality control. Isolated heart mitochondria were treated with Proteinase K to remove cytosolic contaminants and enriched for inner mitochondrial membrane proteins; cytosolic (tubulin), outer membrane protein (VDAC), inner membrane protein (COXIV).

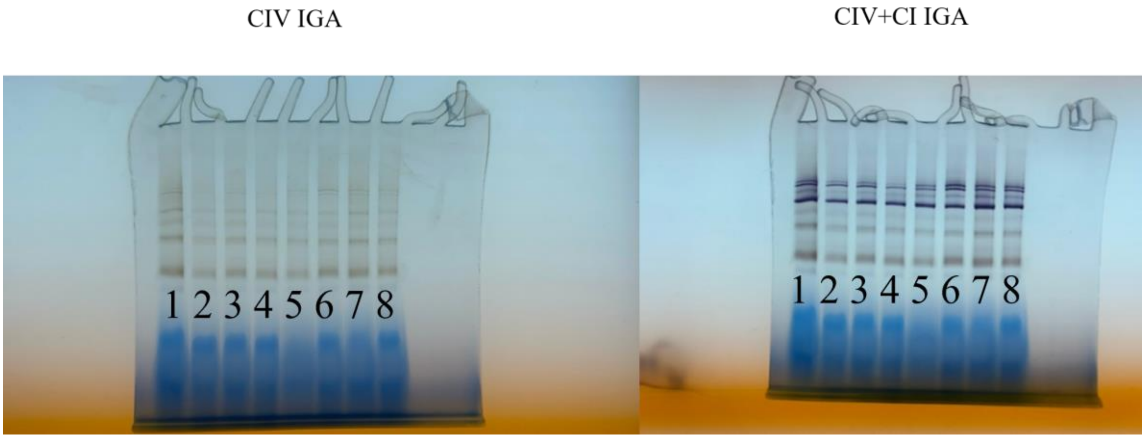

Figure S4. Uncropped images for Figure. 1B-C. CIV IGA, and CIV+CI IGA. Images were taken after overnight incubation and on a phone. Total of 4 biological replicates per group. Sample group and lane assignments: WT 1-4, COX7aH KO 5-8.

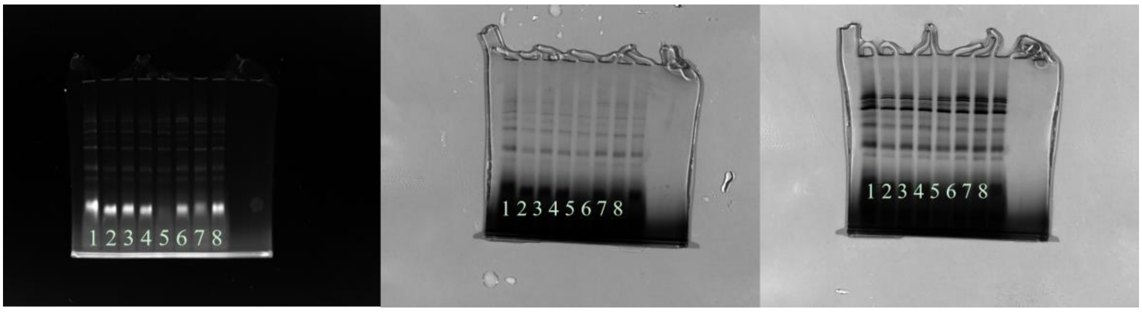

Figure S5. Uncropped images for Figure. 1A-C. Uncropped Coomassie and CIV IGA, and CIV+CI IGA. Images were taken after 1 hour of incubation and on a Chemidoc imager and used for IGA quantification and normalization. Total of 4 biological replicates per group. Sample group and lane assignments: WT 1-4, COX7aH KO 5-8.

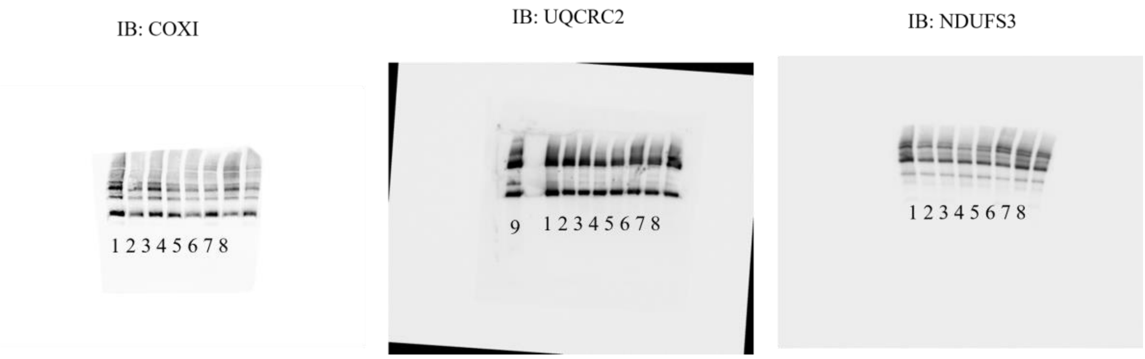

Figure S6. Uncropped images for Figure. 3A-C. Uncropped immunoblots of COXI, UQCRC2, and NDUFS3. Total of 4 biological replicates per group. Sample group and lane assignments: WT 1-4, COX7aH KO 5-8. Lane 9 was a rat heart used as a positive control for the Q-respirasome.

Coomassie Stain Coomassie Stain

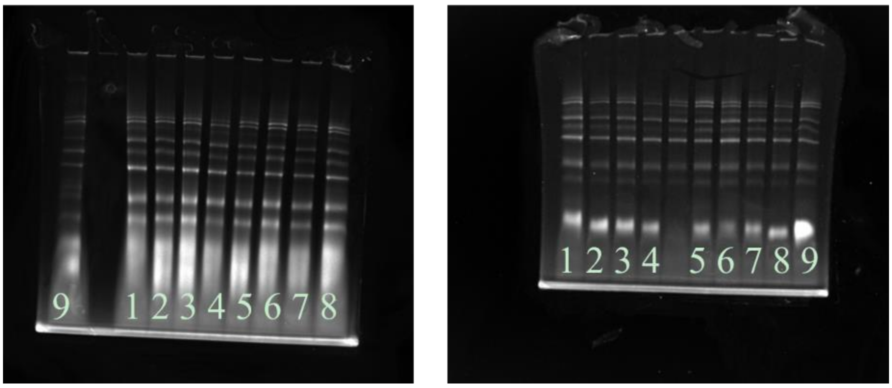

Figure S7. Uncropped images for Figure 3. Uncropped Coomassie blot used for total protein normalization of UQCRC2 (left), COXI (right), and NDUFS3 (right). WT 1-4, COX7aH KO 5-8. Lane 9 was a rat heart used as a positive control for the Q-respirasome.

IB: SCAF1 IB: UQCRC2

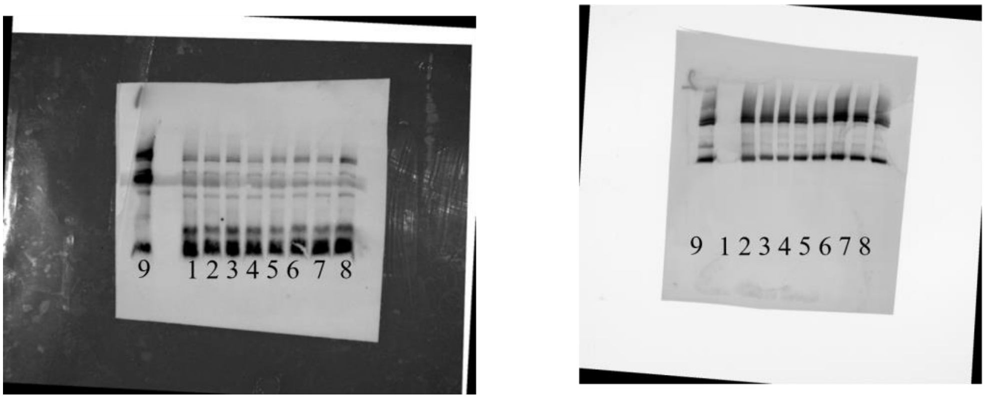

Figure S8. Uncropped images for Supplementary Figure. S2A and B uncropped UQCRC1 and SCAF1. Sample group and lane assignments: WT 1-4, COX7aH KO 5-8. Lane 9 was a rat heart used as a positive control for III_2+IV SC.

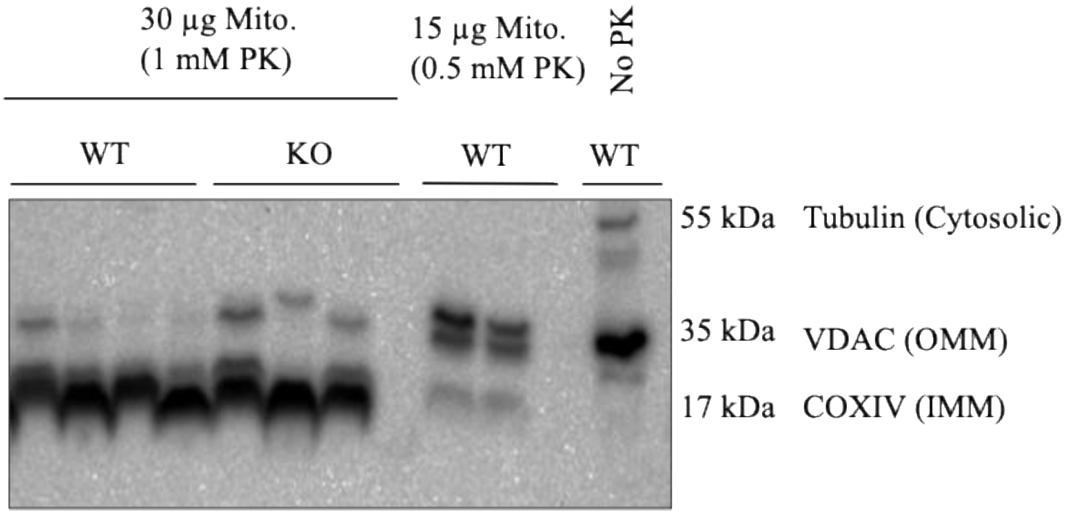

Figure S9. Uncropped images for Supplementary Figure S3.
