# Supplementary figures and images for: Supercomplex Restructuring in Heart Mitochondria of COX7A1-Deficient Mice
Source: Biomolecules. 2025 Aug 22;15(9):1209. doi: 10.3390/biom15091209 (PMC12467167; doi:10.3390/biom15091209)

|                                   | WT-1 | WT-2 | KO-1 | KO-1 |
|-----------------------------------|------|------|------|------|
| IV                                | 1    | 7    | 13   | 19   |
| IV <sub>2</sub>                   | 2    | 8    | 14   | 20   |
| III <sub>2</sub> +IV              | 3    | 9    | 15   | 21   |
| III <sub>2</sub> +IV <sub>2</sub> | 4    | 10   | 16   | 22   |
| SC <sub>1</sub>                   | 5    | 11   | 17   | 23   |
| SC <sub>2</sub>                   | 6    | 12   | 18   | 24   |

Coomassie Stain

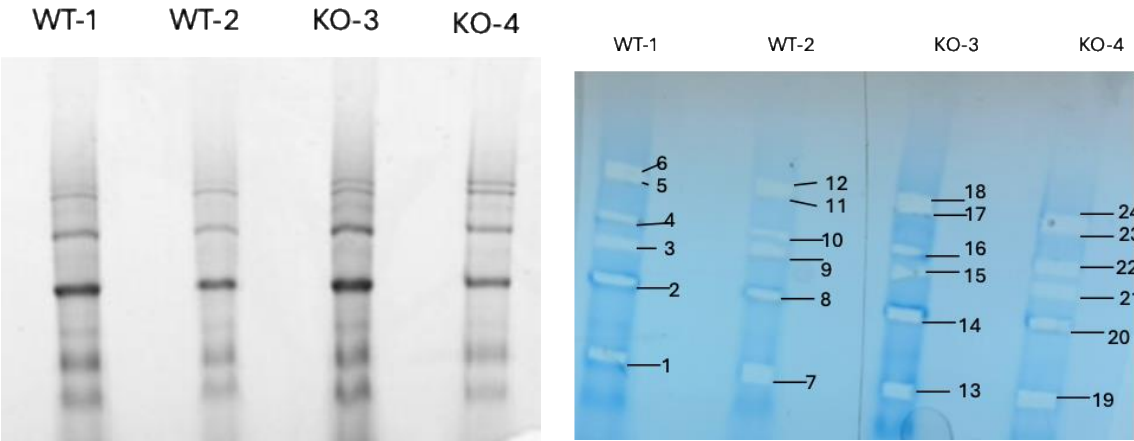

Supplement: Supplementary file 1 [file biomolecules-15-01209-s001.zip › Mass Spec Gel- Annotation.pdf]
